# Case report: Response to tepotinib in Chinese non-small cell lung cancer patients harboring METex14 skipping with varying features

**DOI:** 10.3389/fonc.2024.1383964

**Published:** 2024-07-02

**Authors:** Yan Meng, Weiping Zhou, Chenping Li, Xiangjie Zhou, Xiao Li, Liang Li, Qiye Fu, Jue Huang, Yali Yue, Xuguang Shen, Lijing Yang, Meiqing Wang

**Affiliations:** ^1^ Department of Oncology II, Hainan Cancer Hospital, Haikou, Hainan, China; ^2^ Department of Pharmacy, Boao Super Hospital, Qionghai, Hainan, China

**Keywords:** tepotinib, lung cancer, METex14, case series, MET TKI

## Abstract

MET exon 14 (METex14) skipping is the most reported MET mutation in non-small cell lung cancer (NSCLC) and has been confirmed to respond to MET tyrosine kinase inhibitors (TKI) in clinical trials. While MET TKI tepotinib was recently approved for METex14 skipping NSCLC in China, real-world evidence is limited. We report our experience treating NSCLC patients referred from oncology sites across China with tepotinib in the Boao Lecheng Pilot Zone. Four patients have been prescribed the drug with a median age of 67 years (range, 61–71 years). One patient has concomitant BRAF V600E mutation, and another patient had savolitinib as first line of therapy but discontinued due to hepatotoxicity. Till the end of follow-up, four patients were all on tepotinib therapy, with a median duration of therapy of 19 months. One patient achieved partial response and three achieved stable disease. Three patients had peripheral edema, but all were mild. Our experience showed in real clinical setting, tepotinib had robust and durable clinical activity and a favorable toxicity profile in Chinese patients with METex14 skipping NSCLC. It is the first report on the effectiveness of tepotinib in a patient with both METex14 skipping and BRAF V600E mutations and successful MET inhibitor switch after MET inhibitor-induced liver injury.

## 1 Introduction

Lung cancer is one of the leading causes of cancer morbidity and mortality worldwide (1). Non-small cell lung cancer (NSCLC) accounts for approximately 85% of all diagnosed lung cancer cases globally and is a heterogeneous disease that might be caused by various oncogenic driver mutations (2). Alterations in the mesenchymal-epithelial transition (MET) gene have been recognized as a primary oncogenic driver in NSCLC, and mutations leading to MET exon 14 (METex14) skipping are the most reported oncogenic MET mutations, occurring in around 0.9–4% of patients with NSCLC (3–5).

Several tyrosine kinase inhibitors targeting MET have been evaluated for the treatment of NSCLC patients with METex14 skipping and have shown favorable antitumor activities in clinical trials (6–9). Tepotinib is a highly selective and potent small molecule inhibitor of MET. In the open-label, phase II VISION study, tepotinib was found to deliver robust and durable efficacy in patients with advanced NSCLC with METex14 skipping, as evidenced by an objective response rate (ORR) by independent review of 51.4% and a median duration of response (DOR) of 18.0 months (9). Results of the VISION study have led to regulatory approval of tepotinib in March 2020 in Japan (6), followed by multiple other countries, including the US and EU, for the approval of tepotinib for locally advanced and/or metastatic METex14 NSCLC.

In China, tepotinib was recently approved for the same indication in December 2023, primarily based on data from the Chinese patients in the VISION study. Although the efficacy and safety of tepotinib have been confirmed by the clinical trial, data from real clinical practices are still quite limited. The real-world use of tepotinib in patients of different characteristics and sometimes with suboptimal treatment conditions can provide valuable insights into the effectiveness of the drug and disease management for different patients in real clinical setting.

Hainan Bo’ao Lecheng International Medical Tourism Pilot Zone is a special region of Hainan, China, where certain new drugs that have been approved overseas can be legally prescribed to patients in need after a series of administrative procedures. In the Pilot Zone, patients with indications for the therapy can receive tepotinib under physician supervision in routine clinical settings, free from the restrictions typically imposed by clinical trials. In this paper, we report our experience as healthcare providers in the Pilot Zone in treating NSCLC patients harboring METex14 skipping with tepotinib.

## 2 Methods

For this case series, all patients with locally advanced or metastatic NSCLC who tested positive for METex14 skipping and were treated with tepotinib in the Pilot Zone were included. No other selection criteria were applied for the study purpose. Patients who might benefit from tepotinib after evaluation by their primary physician from oncology sites across China can be referred to Hainan Pilot Zone for tepotinib prescription. The evaluation includes biomarker testing, histology, and any other examinations the physician deems necessary for decision-making. Patients would then travel to Hainan Cancer Hospital in Haikou, Hainan for formal evaluation performed by a dedicated local physician. The evaluation includes tumor assessment by computed tomography (CT) or magnetic resonance imaging (MRI) and a series of examinations routinely performed for hospitalized patients, including electrocardiogram, blood sample collection for hematology, biochemistry, and physical examination. Biomarker testing and histology would not be repeated. Based on the evaluation results, the physician would decide if tepotinib can be prescribed to the patient. All the evaluation were performed within 10 days prior to the initiation of tepotinib.

Patients ready for tepotinib therapy would need to travel to the Boao Super Hospital located in the Pilot Zone for tepotinib prescription and administration, accompanied by the physician of Haikou. In the first week of tepotinib administration, patients were hospitalized for safety considerations. After that, they were allowed to take prescriptions which can last up to 3 months out of the Pilot Zone, back to their primary physician. The prescriptions issued to the patients are closely monitored by the issuing hospital in the Pilot Zone (Boao Super Hospital) through a data collection system to track medication usage. Following the start of tepotinib, patients had follow-up visits for tumor assessment either at the oncology site of the patients’ primary physician or at the Hainan Cancer Hospital by the physician there. Radiographic responses to MET inhibition were evaluated using Response Evaluation Criteria in Solid Tumors (RECIST), version 1.1. The findings from the tumor assessment together with imaging data were exchanged between the primary physician and the Haikou physician after each assessment, and the medical decision regarding NSCLC treatment were collaboratively made by the two physicians. Till now, no major disagreement occurred between the two physicians. For adverse event monitoring, patients were suggested to come back to either their primary physician or the Haikou physician every 2 weeks (± 3 days) for the first month and every 4 weeks (± 3 days) thereafter for safety assessment, as part of the routine medical care requirement when receiving a drug from the Pilot Zone.

For this case series, a retrospective systematic chart review was performed by physicians involved in the medical care of the patients included, and demographic and clinical information were extracted from the electronic medical records.

## 3 Results

Starting from January 2022, when tepotinib was first introduced to the Pilot Zone, a total of four patients have been prescribed the drug, and these patients were followed through November 2023. **Table 1** summarizes the demographic and clinical characteristics of the four patients. Three of the patients were men and the median age at admission was 67 years (range, 61 to 71 years). Three patients had stage IVa adenocarcinoma, and one had IIIc adenocarcinoma. Two patients were treatment-naïve prior to MET therapy, and the other two were treated with pemetrexed and nedaplatin chemotherapy and savolitinib, respectively as first line.

**Table 1:** Demographic and clinical characteristics of patients and response to tepotinib therapy.

Case	Age	Sex	Stage	Histology	Site of metastasis	Biopsy type	Assay	METex14 mutation	Other driver mutation	Duration of tepotinib therapy	Response
1	65	Female	IVa	Adenocarcinoma	The other lung	Tissue biopsy	NGS	c.3028G>A	BRAF V600E, TP53, and PDGFRA MT mutations	19 months, ongoing	SD
2	71	Male	IVa	Adenocarcinoma	Pleura, mediastinum, lymph node	Tissue biopsy	ARMS	Unknown	No	20 months, ongoing	PR
3	69	Male	IVa	Adenocarcinoma	Lymph nodes, bone	Tissue biopsy	NGS	c.3028G>C (p.D1010H)	No	19 months, ongoing	SD
4	61	Male	IIIc	Adenocarcinoma	Lymph nodes, the other lung	Liquid biopsy	NGS	c.2942–11_2966del	TP53	3 months, ongoing	SD

ARMS, amplification-refractory mutation system; METex14, mesenchymal-epithelial transition exon 14; NGS, next-generation sequencing; PR, partial response; SD, stable disease.

Till the end of follow-up, four patients were all on tepotinib therapy, with a median duration of therapy of 19 months (range, 3 to 20 months). One patient achieved partial response (PR) and three achieved stable disease (SD) at the end of follow-up. From the safety perspective, three patients had peripheral edema, but all were mild.

### 3.1 Case 1: coexistence of METex14 with BRAF V600E

**Figure 1:**
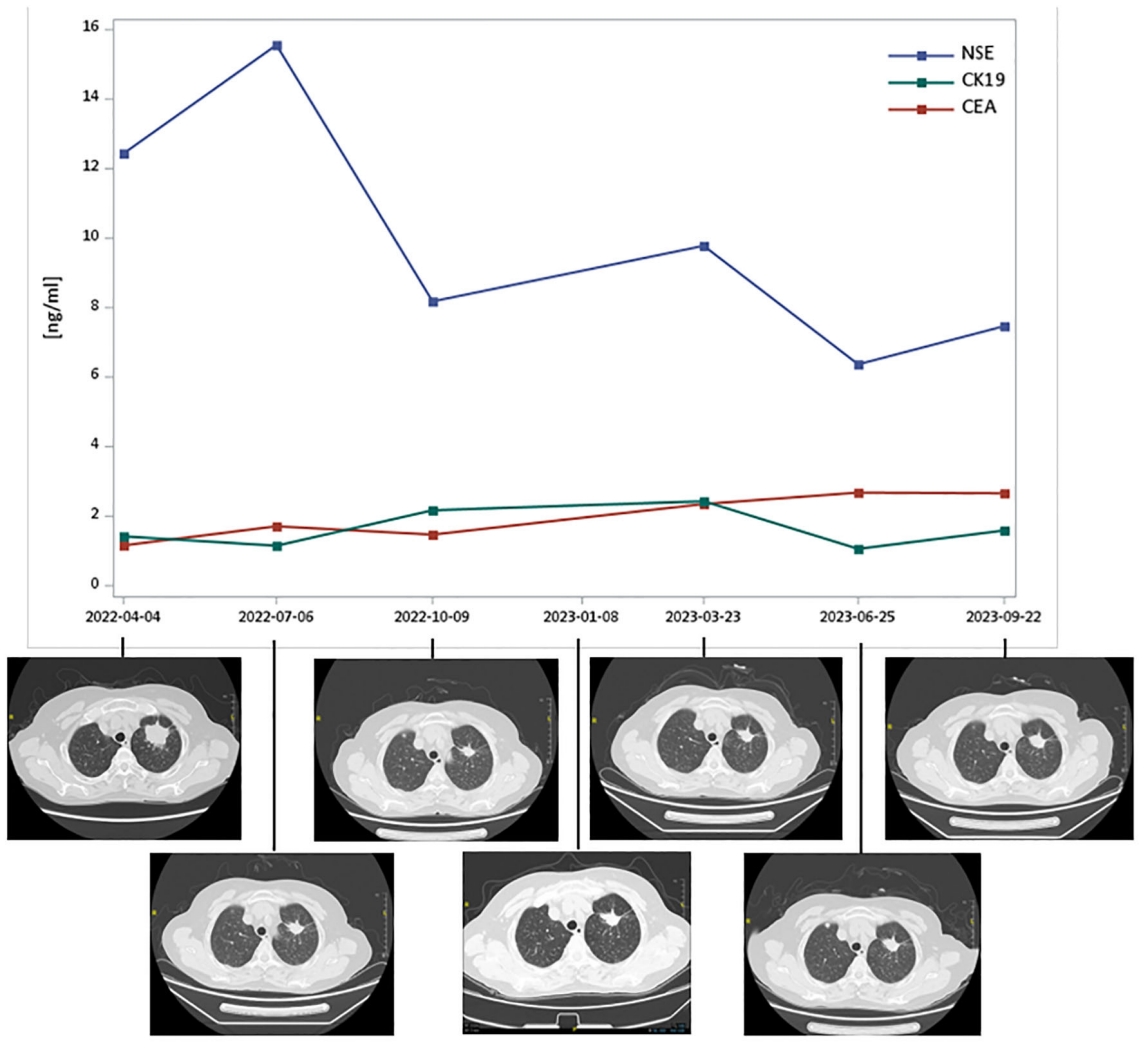
Tumor markers and thoracic computed tomography scans of Case 1 over MET TKI treatment. CEA, carcinoembryonic antigen; CK19, cytokeratin 19; NSE, neuron-specific enolase; TKI, tyrosine kinase inhibitors. Tepotinib therapy started in April 2022 and follow-up CT scan at 3-, 6-, 9-, 11-, 14- and 17-month suggested that lesions were stable.

The patient is a 65-year-old woman, never smoker, who complained of right sided facial numbness and numbness in right arm for half a month. Brain CT identified a cerebral aneurysm of right middle cerebral artery, which was subsequently treated by aneurysm clipping surgery. Chest CT performed at the same time (in December 2021) detected a left upper lobe mass (2.3x1.6cm) with multiple, small bilateral pulmonary nodules, and a needle biopsy of left upper lobe lesion confirmed well-differentiated adenocarcinoma, with EGFR (+), P63 (-), Ki-67 10%+, CK7 (+), p53 weak, Napsin A (+), TTF-1 (+), ALK (-). Next-generation sequencing (NGS) suggested METex14 skipping (c.3028G>A), BRAF V600E (c.1799T>A), TP53, and PDGFRA MT mutations. The patient presented to our institution for tepotinib treatment starting from April 2022. After a week of tepotinib (450mg, orally, daily), bilateral lower limb edema (grade 2) was reported, which was relieved after oral administration of diuretics. Follow-up CT scan at 3-, 6-, 9-, 11-, 14- and 17-month suggested that lesions were stable (**Figure 1**). Tumor marker neuron-specific enolase (NSE) showed a declining trend over treatment and carcinoembryonic antigen (CEA) and cytokeratin 19 (CK19) remained within the normal range (**Figure 1**). The patient was still alive and continued on tepotinib therapy with clinically and radiologically stable disease at the end of follow-up.

### 3.2 Case 2: significant reduction in tumor size

**Figure 2:**
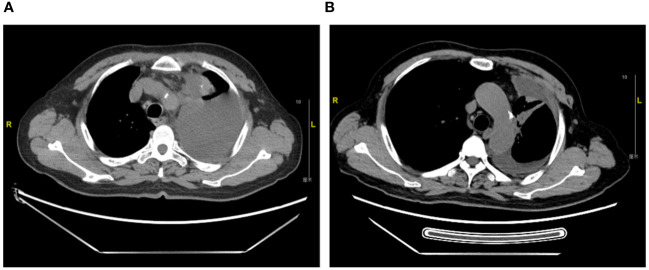
Thoracic computed tomography scans of Case 2. **(A)**. Diagnosis of metastatic NSCLC in March 2022 **(B)**. Partial response with a significant decrease in size of left upper lobe lung mass at 6-month follow up in September 2022.

The patient is a 71-year-old previously smoking man, presented with a chief complaint of chest distress. In February 2022, the patient underwent chest CT in a local clinic, which revealed left pleural effusion. He was then referred to a cancer hospital for further examination and CT scan showed neoplastic lesions in the left upper lobe with possible pleural, mediastinal and left hilar lymph node metastases. Adenocarcinoma was subsequently confirmed by a needle biopsy of the left lung mass and immunohistochemistry testing suggested PD-L1 (TPS 25%). Tepotinib therapy (450mg, orally, daily) was initiated in March 2022 after the detection of METex14 skipping by amplification-refractory mutation system (ARMS). Response to tepotinib was rapid, as observed by significant decrease in size of the left lung mass at the first post-tepotinib tumor evaluation already. Three-, 6- and 9-month follow-up CT scan continuously showed partial response from the baseline scan (**Figure 2**). Tepotinib was overall well tolerated, with the main adverse event being grade 2 edema on bilateral upper and lower limb, which was relieved after oral administration of diuretics. Hematology and biochemistry monitoring showed no abnormal results. The patient was still on tepotinib therapy by the end of follow-up.

### 3.3 Case 3: good tolerance for patient with multiple comorbidities

A 69-year-old man, former smoker, presented with a chief complaint of intermittent cough and expectoration for over 2 months. PET-CT performed in February 2022 revealed bilateral lung masses, multiple lymph node metastases in the bilateral mediastinum, hilum, neck, and supraclavicular region, and increased local metabolism in the 7th thoracic vertebral body and sacral margin, suggesting neoplastic lesions. Biopsy of neck lymph node confirmed lung adenocarcinoma metastasis. Genetic testing detected METex14 skipping mutations (c.3028G>C (p.D1010H)). The patient received pemetrexed and nedaplatin chemotherapy for one cycle at a local oncology site, which however, was poorly tolerated. He was then initiated on tepotinib (450mg, orally, daily) at our institution in April 2022 and closely monitored for safety. Follow-up CT scan at 3-, 5-, and 8-month suggested that lesions were stable. Overall, tepotinib was very well tolerated by the patient, with only mild limb edema (grade 1) appearing after five months of treatment, with no need for medical intervention. The patient remained progression free on tepotinib therapy by the end of follow-up.

Medical history showed that the patient had acute myocardial infarction in December 2021 and was treated with drug-eluting stent in the proximal/middle segment of anterior descending artery. After that, the patient has been on clopidogrel (75mg, daily), indobufen (10mg, twice daily), antiplatelet, sacubitril-valsartan (50mg, twice daily), and rosuvastatin (10mg, every night). In addition, the patient has been diagnosed with hypertension and treated with metoprolol (47.5mg, daily) since 2018, with the highest blood pressure record of 205/60mmHg. The patient also has a history of cerebral infarction.

### 3.4 Case 4: MET inhibitor switch after potential MET inhibitor-induced liver injury

**Figure 3:**
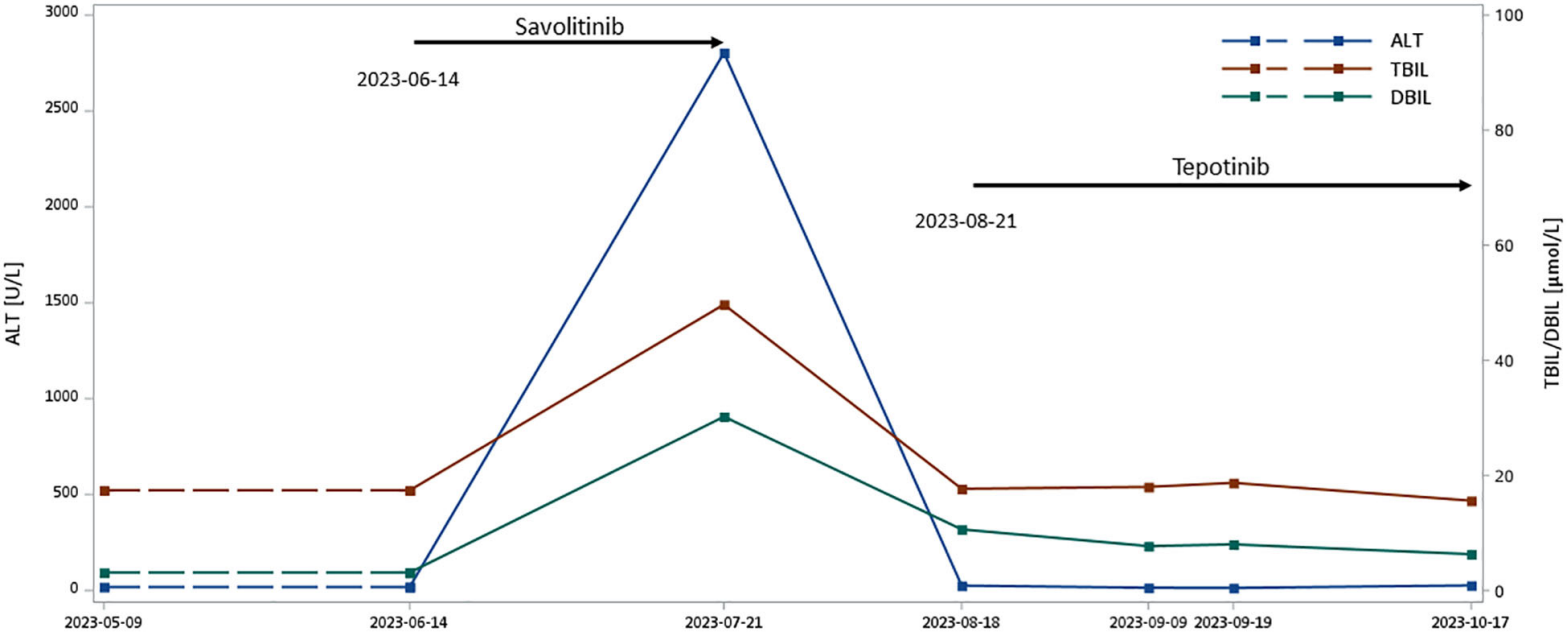
Liver function of Case 4 over MET TKI treatment. ALT, alanine transaminase; DBIL, direct bilirubin; TBIL, total bilirubin; TKI, tyrosine kinase inhibitors Liver function results immediately prior to savolitinib initiation are not available, and thus results from 9 May 2023 were taken as the reference liver function status before savolitinib therapy. Lines between 9 May 2023 and 14 June 2023 are thus displayed as dashed lines.

The patient is a 61-year-old previously smoking man. In a routine health check, the patient underwent a chest CT, which identified ground-glass nodular shadow in the left upper lobe. Follow-up PET-CT at a local hospital revealed significantly larger mass in the basal segment of the right lower lobe than previous CT scans performed at the same hospital, with increased metabolism, and multiple subsolid nodules in both lungs and subpleural, suggesting neoplastic lesions. Biopsy of lymph node confirmed lung adenocarcinoma and immunohistochemistry testing showed CK7 (+), P40 (-), TTF-1 (+), Ki-67 (index 80%), and P53 (+). NGS detected METex14 skipping (c.2942–11_2966del). The patient was first treated by savolitinib (600mg, orally, once daily) at the local hospital starting from June 2023 for one month, during which he had significantly elevated level of alanine aminotransferase (ALT) of 2804U/L and increased total and direct bilirubin (**Figure 3**), accompanied by rash, mouth ulcer and fever over the course. Savolitinib was then discontinued due to hepatotoxicity, and by the time of discontinuation, the disease was stable.

Considering the potential effectiveness of MET inhibitors, the patient’s primary physician referred him to our institution for tepotinib. After one month from savolitinib discontinuation and a re-evaluation of ALT, which dropped back to 25U/L (**Figure 3**), the patient was initiated on tepotinib (450mg, orally, daily) in August 2023. A two-month follow-up liver function test in October 2023 suggested normal ALT and bilirubin levels (**Figure 3**), and no adverse event was observed over the period. The patient was still alive and continued on tepotinib therapy with clinically and radiologically stable disease at the end of follow-up.

## 4 Discussion

In this paper, we present the four NSCLC patients with METex14 skipping treated with tepotinib in the Pilot Zone. Tepotinib therapy was chosen for these patients due to the presence of METex14 skipping alterations and its superior efficacy in trials compared to savolitinib, the only other available MET TKI in China at that time (10). All patients were older adults and were diagnosed with adenocarcinoma. Genetic mutations were confirmed by NGS in three patients and ARMS in the other patient. Demographic and clinical characteristics of our patients are in consistent with those from previous reports, in which METex14 skipping was found to frequently occur in older adults, with a reported median age ranging from 65 to 76 years and commonly in adenocarcinoma (11).

In NSCLC, METex14 skipping alterations typically do not co-occur with other primary oncogenic drivers, such as EGFR, ALK, ROS1, BRAF, KRAS, ERBB2, or RET (12–14). At our institution, we observed a patient with both METex14 skipping and BRAF V600E mutations, which rarely co-exist in previous literature. In such a case, patient might respond to either MET tyrosine kinase inhibitors (TKI) or BRAF/MEK dual inhibitors therapy, as the mechanism behind concomitant alterations remains to be elucidated. A phase II study of patients with BRAF V600E-mutant metastatic NSCLC reported a patient with concomitant METex14 skipping, who was treated by trial regimen – dabrafenib 150 mg twice daily plus trametinib 2 mg once daily orally after progression on platinum-based chemotherapy (15). The patient had partial response with a progression-free survival of 10.2 months and an overall survival of 18.2 months. In our case, the patient treated by tepotinib was still alive with clinically and radiologically stable disease by the end of follow-up, which had already lasted 19 months. The decision to start with drug targeting METex14 skipping was made based on the patient’s preference and medical history of cerebral aneurysm and the corresponding higher requirements for drug safety, as nervous system adverse reactions, such as dizziness and headache, and vascular adverse reactions, such as hypertension and subarachnoid hemorrhage, have been reported in patients treated with BRAF inhibitors (15). Additionally, in the phase II study of dabrafenib plus trametinib, nearly half of the patients experienced at least one grade 3–4 event (16). The treatment of this patient suggests that the inhibitor targeting METex14 skipping might be an effective treatment choice in patients with concomitant METex14 skipping and BRAF V600E mutations, and this can be particularly relevant when drug safety is a special concern. A possible explanation is the activation of c-Met could further activate multiple signaling pathways involved in cell survival, motility, and proliferation, including RAS/ERK/MAPK pathway, and thus MET TKI could block MET-dependent downstream signaling (17). Further studies would be needed to investigate the potential role of co-occurring genomic alterations in drug resistance and tumor response.

In patients of our institution, tepotinib showed durable clinical activity as witnessed by obvious tumor shrinkage in one patient and stable disease in the other three patients over the period of 1.5 years. Of note, two of the three patients with stable disease also experienced tumor shrinkage, although it did not meet the criteria for a partial response. The real-world effectiveness of tepotinib is in line with efficacy results of the phase II VISION study, in which 313 patients with advanced NSCLC harboring METex14 skipping were treated by tepotinib 500 mg once daily (9). Over a median follow-up of 32.6 months, ORR by independent review was 51.4% (95% confidence interval [CI], 45.8%-57.1%) with a median DOR of 18.0 (95% CI, 12.4–46.4) months. Median progression-free survival and overall survival were 11.2 (95% CI, 9.5–13.8) months and 19.6 (95% CI, 16.2–22.9) months, respectively. In 149 previously treated patients, ORR achieved 45.0% (95% CI, 36.8%-53.3%) with a median DOR of 12.6 (95% CI, 9.5–18.5) months (9). Real-world experience further confirmed the durable response to tepotinib in patients with widely metastatic lung adenocarcinoma.

Tepotinib was very well tolerated by the patients treated at our institution, with mild edema as the only adverse event in three patients. No serious adverse event was observed. Even in the patient with a history of acute myocardial infarction and multiple cerebrocardiovascular comorbidities, tepotinib showed a favorable safety profile with several concomitant medications for cerebrocardiovascular comorbidities. Data from our real-world cases is consistent with the results of the VISION study, in which peripheral edema was the most common treatment-related adverse event occurring in around two-thirds of the patients (9). Beyond that, our case suggests that tepotinib seems to be safe together with common cardiovascular medications in elderly patients with multiple cerebrocardiovascular comorbidities and no further unexpected adverse events occurred. Additionally, hepatotoxicity could be an issue that needs to be closely monitored. In the multicenter, phase II study of savolitinib, 10% (n=7) of patients experienced treatment-related increased ALT of grade 3 or more (18). In our case, we report, for the first time, a patient who successfully switched MET inhibitors after potential savolitinib-induced liver injury. The patient’s ALT levels returned to normal after the discontinuation of savolitinib and remained within the normal range during three months of tepotinib therapy, which suggests that another MET inhibitor might still be considered even after MET inhibitor-induced liver injury. This further indicates that while MET inhibitors of the same class might share the same action mechanism, they do not necessarily have identical toxicity profiles due to the potential differences in metabolic patterns.

Some limitations should also be noted when interpreting the results. First, we only report a small number of patients treated at a single institution, which might not fully represent the effectiveness and safety of tepotinib in diversified clinical settings, despite the unique characteristics of the individual patients included. Another limitation relates to the fact that all these patients are still on treatment, so the final outcomes remain to be updated.

Our experience of prescribing tepotinib in the Hainan Pilot Zone showed that in real clinical setting, tepotinib had robust and durable clinical activity and a favorable toxicity profile in Chinese patients with METex14 skipping NSCLC. We also for the first time report the effectiveness of tepotinib in a patient with both METex14 skipping and BRAF V600E mutations and successful MET inhibitor switch after potential MET inhibitor-induced liver injury.

## Data availability statement

The datasets used and/or analyzed during the current study are available from the corresponding author on reasonable request. NGS raw data are stored at the institution of the patients’ primary physicians and are not available from the authors. Requests to access these datasets should be directed to corresponding author.

## Ethics statement

Ethical approval was not required for the study involving humans in accordance with the local legislation and institutional requirements. Written informed consent to participate in this study was not required from the participants or the participants’ legal guardians/next of kin in accordance with the national legislation and the institutional requirements. Written informed consent was obtained from the individual(s) for the publication of any potentially identifiable images or data included in this article.

## Author contributions

YM: Conceptualization, Formal analysis, Investigation, Writing – original draft, Writing – review & editing. WZ: Formal analysis, Investigation, Writing – original draft, Writing – review & editing, Validation. CL: Investigation, Writing – review & editing. XZ: Investigation, Writing – review & editing. XL: Investigation, Writing – review & editing. LL: Investigation, Writing – review & editing. QF: Investigation, Writing – review & editing. JH: Investigation, Writing – review & editing. YY: Investigation, Writing – review & editing. XS: Investigation, Writing – review & editing. LY: Investigation, Writing – review & editing. MW: Conceptualization, Formal analysis, Investigation, Supervision, Writing – original draft, Writing – review & editing.
